# Factors Associated with Severe Deliberate Self-Harm among Chinese Internal Migrants

**DOI:** 10.1371/journal.pone.0080667

**Published:** 2013-11-19

**Authors:** Yuanyuan Xiao, Naiqing Zhao, Min Yu, Ming Zhao, Jieming Zhong, Weiwei Gong, Ruying Hu

**Affiliations:** 1 School of public health, Fudan University, Shanghai, China; 2 Zhejiang provincial center for disease control and prevention, Hangzhou, Zhejiang, China; Pennsylvania State University, United States of America

## Abstract

**Background:**

Studies on mental health status of Chinese internal migrants are sparse albeit desperately needed. Deliberate self-harm (DSH) is intimately related to mental disorders, especially depression based on literatures. The major aim of this study is to explore associated factors of severe DSH among Chinese internal migrants.

**Methods:**

Totally 426 DSH migrants identified by a provincial injury sentinel surveillance system between the year 2005 and 2010 were analyzed. Descriptive statistics were used to depict general characteristics of those cases. Chi-square test was used to explore inter-stratum distributive differences of self-harm severity by multiple factors. Logistic regression model was employed to estimate associations between severe self-harm and factors of interest.

**Results:**

Among all identified DSH migrants, females took the majority (66.2%), younger individuals accounted for nearly two-thirds of all study subjects. Based on logistic regression model fitting result, age and preceding alcohol drinking were significantly related to self-harm severity, whereas residence place and self-harm method only showed associations with severe DSH in females.

**Conclusions:**

Among Chinese internal migrants, older ones and females who reside in county areas could be more vulnerable to severe self-harm, population-based studies which focusing on the characteristics and risk factors of mental health well-being among Chinese internal migrants are urgently warranted.

## Introduction

The lasting internal migration in China probably can be called the most magnificent population reshuffling in modern history. Thanks to the loosening of household registration regulation ever since the implementation of “opening-up policy” in the late 1970s and early 1980s [1], along with the modernization of agriculture which resulted in a huge surplus of labor in rural regions [2], lots of inlanders keep swarming into mega cities, especially those reside in the southern and eastern coastal areas of China during the past three decades. According to the 6^th^ National Population Census data, by the end of the year 2010, there were more than 221 million internal migrants in China, over 80 percent increase in amount when compared with the inception of 21^st^ millennium [3], yet a continuous growth is expected in the near future.

In China, internal migration usually occurs without a change of *hukou* (household registration) status. To a great extent, *hukou* status decides one's full access to employment, medical insurance, housing stipend, social welfare and education within the registration area [4]. In this instance, internal migrants are often labeled as “vulnerable individuals” in host cities [5]. Ever since the late 1990^th^, studies which focused on health issues of internal migrants started to burgeon. But nearly all of them were focusing on physical health, like infectious diseases (such as AIDS and tuberculosis) and reproductive health of women [6], whereas studies on psychological well-being were scarce. However, some existing studies in this field had already disclosed a disturbing trend: the overall mental health status of internal migrants is alarming, especially the prevalence of depression. For example, one study which carried out in the capital city of an inland province (Sichuan), Qiu *et al* found that 23.7% of 1,180 internal migrants reported depressive symptoms [7], and another study which implemented in an economically prosperous coastal city of China, among 4,088 internal migrants, a comparable 21.4% reported depressive symptoms [8].

Depression is closely related to deliberate self-harm (DSH). According to western literatures, about two-thirds DSH patients are suffering from depression, simultaneously [9], [10]. Take the large size of population and high prevalence of depressive symptoms into consideration, DSH behaviors among Chinese internal migrants might be more common than that in general population. In Chinese culture, mental illnesses are often attached to stigma [11], thus usually DSH individuals won’t seek help from health facilities unless under urgent situations. Therefore, the predominant part of light DSH cases remains undetectable. However, considering of the fact that severe DSH usually results in much more devastating outcomes such as disabilities or even death, and is more intimately related to mental health distresses rather than impulsion [12], it is of a much greater intervention importance than light self-inflicted injuries. The major aim of our study is to investigate self-harm behaviors among Chinese internal migrants, by looking into the risk factors that associated with severe DSH.

## Method

### Case collection

This study was conducted in Zhejiang province, an economically vibrant coastal region in China, and one of the most favorite destinations to internal migrants. According to Zhejiang provincial bureau of statistics, by the end of the year 2010, there were about 11.8 million internal migrants in Zhejiang, accounted for over one-fifth of total residents [13].

Self-harm internal migrants were selected from the database of *Zhejiang provincial hospital-based injury surveillance system* between the year 2005 and 2010. *Zhejiang provincial hospital-based injury surveillance system* is a sentinel surveillance system which was established in the year 2004. Altogether 28 hospitals from 9 counties were chosen as reporting institutions. Related information of each firstly-diagnosed injury patient was collected and reported by doctors in emergency departments. Written consent was acquired before data collection. For completely sober patients, the written consent was provided by patients themselves. For children patients, the written consent was provided by their parents or guardians. For patients who were unconscious, both the written consent and injury information were provided by one relative or close friend, who was also familiar with the whole incident. The protocols of the surveillance program and this study were approved by institutional review board of Zhejiang provincial center for disease control and prevention.

### Case definition

Self-harm internal migrants were defined as: 1) *Hukou* (household registration) status was either “other provinces except Zhejiang ” or “other city or county of Zhejiang”; 2) 15 to 65 years old; 3) not a student; 4) reported as “self-inflicted injuries”. The main reason to exclude migrant students from the final analytical sample is that, usually their purpose of migration is not economically motivated, which is contrast to the majority of Chinese internal migrants.

### Variables and definitions

Age was measured by year, and further divided into 3 subgroups: 15 to 29 years, 30 to 44 years, and 45 to 65 years. Season of self-harm was defined as: spring (March to May), summer (June to August), autumn (September to November), and winter (December to February). Occupation categories were: temporary manual worker, salaried manual worker, farmer or fisherman, commercial service, self-employed, unemployed, and unspecified occupation. Self-harm methods included: fall, blunt force, stabbing/cutting, poisoning, and other causes. Self-harm severity was measured by outcome: light self-harm cases were those who “heading back home after simple medical disposition” or went through “medical observation”, while severe self-harm cases were those who either “hospitalized”, or “transferred to another hospital”, or “died”. Underlying causes of injury were enumerated as: intra-family conflict, physical ailments, mental disorder or impulsion, and other causes (which included: financial setback, legal implication, death of relative, maltreatment, and unspecified causes). Residence places were categorized into “city” and “county”.

### Statistical analysis

Descriptive analysis was used to depict general characteristics of self-harm internal migrants. Inter-subgroup differences on self-harm severity were examined by Chi-square test. Then, logistic regression model was fitted, by taking self-harm severity as dependent variable, and significant variables based on Chi-square tests as independents.

All statistical analyses were preformed by STATA (Version 12.0; Stata cooperation; College Station, Texas, USA).

## Results

### Characteristics of DSH migrants

From the year 2005 to 2010, altogether 547 self-harm internal migrants were identified. After went through quality review, 121 of them were excluded because of incompleteness of vital information, finally we got 426 cases to analyze. Among those cases, females took the majority (66.2%), younger individuals who aged between 15 to 29 years accounted for nearly two-thirds of all subjects. Temporary and salaried manual worker were the predominant professions, with a combined proportion of 70 percent. About 80 percent subjects were resided in cities. DSH cases were evenly distributed in four seasons. Over 70 percent cases chose either stabbing/cutting or poisoning to hurt themselves. Severe DSH cases accounted for one-third of all subjects (Table 1).

**Table 1:** Characteristics of identified DSH migrants, 2005–2010, Zhjiang, China.

Characteristics	N	%
Gender		
Male	144	33.8
Female	282	66.2
Age		
15–29 years	261	61.3
30–44 years	143	33.6
45–65 years	22	5.16
Occupation		
Temporary manual worker	187	43.9
Salaried manual worker	111	26.1
Commercial service	34	8.0
Other occupations§	42	9.8
Unemployed	52	12.2
Residence place		
City	337	79.1
County	89	20.9
Season of self-harm		
Spring	112	26.3
Summer	122	28.6
Autumn	82	19.3
Winter	110	25.8
Self-harm method		
Stabbing/cutting	167	39.2
Poisoning	140	32.9
Other methods¶	119	27.9
Self-harm severity		
Light	279	65.5
Severe	147	34.5

§ include: farmer or fisherman, self-employed and unspecified occupation.

¶ include: fall, blunt strike, animal bite, burn or scald, suffocation or drowning and unspecified methods.

### Distributive features of self-harm severity

Gender difference in self-harm severity was significant, as the proportion of severe self-harm was much higher among male cases. There was a palpable trend that along with age increase, the proportion of severe self-harm was increasing dramatically, for example, among cases aged 15 to 29 years old, the proportion of severe self-harm was 26.8%, whereas among cases aged 45 to 65 years old, such proportion was 63.6%, instead. Residence place was also significantly associated with self-harm severity, as the proportion of severe self-harm was much higher among DSH migrants who resided in county areas. Preceding alcohol intake was an associated factor, too. A greater proportion of severe self-harm was discovered among subjects who did not take alcohol before the implementation of self-harm behaviors (Table 2).

**Table 2:** Distributive features of self-harm severity among identified DSH migrants, 2005-2010, Zhejiang, China.

Factors	Self-harm severity		?^2^ value
	Light N (%)	Severe N (%)	
Gender			4.9*
Male	84 (58.3)	60 (41.7)	
Female	195 (69.2)	87 (30.8)	
Age			20.9^**^
15–29 years	191 (73.2)	70 (26.8)	
30–44 years	80 (55.9)	63 (44.1)	
45–65 years	8 (36.4)	14 (63.6)	
Employment status†			0.8
Employed	242 (64.7)	132 (35.3)	
Unemployed	37 (71.2)	15 (28.8)	
Residence place			9.5^**^
Prefecture-level city	233 (69.1)	104 (30.9)	
County	46 (51.7)	43 (48.3)	
Season of self-harm			1.7
Spring	71 (63.4)	41 (36.6)	
Summer	76 (62.3)	46 (37.7)	
Autumn	57 (69.5)	25 (30.5)	
Winter	75 (68.2)	35 (31.8)	
Self-harm method			31.4^**^
Stabbing/cutting	136 (81.4)	31 (18.6)	
Poisoning	80 (57.1)	60 (42.9)	
Other methods¶	63 (52.9)	56 (47.1)	
Underlying cause			8.9*
Intra-family conflict	171 (64.5)	94 (35.5)	
Mental disorder/impulsion	87 (73.7)	31 (26.3)	
Other causes	21 (48.8)	22 (51.2)	
Preceding alcohol intake			7.4^**^
Yes	67 (77.9)	19 (22.1)	
No	212 (62.3)	128 (37.7)	

* *p*<0.05 ^**^ *p*<0.01.

† “employed” include: temporary manual worker, salaried manual worker, farmer or fisherman, commercial service, self-employed and unspecified occupation.

¶ include: fall, blunt strike, animal bite, burn or scald, suffocation or drowning and unspecified methods.

### Model fitting results

During model fitting process, we initially put all significant variables based on Chi-square tests (gender, age, residence place, self-harm method, underlying cause, and preceding alcohol intake) as independents into the logistic regression model, but fitting result was not ideal (goodness-of-fit χ^2^ = 124.05, *df* = 90, *p* = 0.01). Then we examined possible interactions between independents, at the setting significance level (*p*<0.10), we detected out interactions between gender and two other variables (residence place and self-harm method). After putting those interactions into the original model, underlying cause was not significantly associated with self-harm severity anymore, so we dropped it to construct the final regression model. After dropping underlying cause, partial-regression coefficients of other independents showed minute changes, fitting result suggested that this final model was satisfactory (goodness-of-fit χ^2^ = 48.02, *df* = 54, *p* = 0.28).

The estimates of the final logistic regression model were enumerated in Table 3: compared with DSH migrants who aged between 15 to 29 years old, the odds of severe self-harm in those aged 45 to 65 years old was 3.44 (95% CI: 1.30, 9.15) times; preceding alcohol drinking was inversely related to severe self-harm, with an odds ratio of 0.40 (95% CI: 0.22, 0.74); although the main effect of gender indicated that compared with females, the odds of severe DSH was 8.55 (95% CI: 3.54, 20.67) times among males, after taking those two identified interactions into consideration, in every possible combination of residence place and self-harm method, there was no significant difference in the odds of severe DSH between males and females, and the male-to-female odds ratio ranged from 0.28 (95% CI: 0.01, 6.49) to 2.18 (95% CI: 0.30, 15.80).

**Table 3:** Estimates of the final logistic regression model, 2005-2010, Zhejiang, China.

Independent variables	Coefficients (95% CI)	Adjusted OR (95% CI)	Wald Statistic
Gender			
Female	-	1	
Male	2.15 (1.26, 3.03)	8.55 (3.54, 20.67)	4.8**
Age			
15–29 years	-	1	
30–44 years	0.49 (0.20, 0.96)	1.64 (1.23, 2.62)	2.1*
45–65 years	1.24 (0.26, 2.21)	3.44 (1.30, 9.15)	2.5*
Place of residence			
City	-	1	
County	1.10 (0.44, 1.76)	3.01 (1.55, 5.84)	3.3**
Method of self-harm			
Stabbing/cutting	-	1	
Poisoning	1.54 (0.80, 2.29)	4.68 (2.22, 9.87)	4.1**
Other methods¶	2.06 (1.25, 2.87)	7.82 (3.28, 17.59)	5.0**
Preceding alcohol intake			
No	-	1	
Yes	−0.91 (−1.52, −0.30)	0.40 (0.22, 0.74)	−2.9**
Gender × Residence place			
Female × city	-	1	
Male × county	−1.37 (−2.48, −0.27)	0.25 (0.08, 0.77)	−2.4*
Gender × Self-harm method			
Female × Stabbing/cutting	-	1	
Male × Poisoning	−1.97 (−3.16, −0.79)	0.14 (0.04, 0.45)	−3.3**
Male × Other causes	−2.06 (−3.21, −0.91)	0.13 (0.04, 0.40)	−3.5**

* *p*<0.05.

** *p*<0.01.

¶ include: fall, blunt strike, animal bite, burn or scald, suffocation or drowning and unspecified methods.

**Figure 1:**
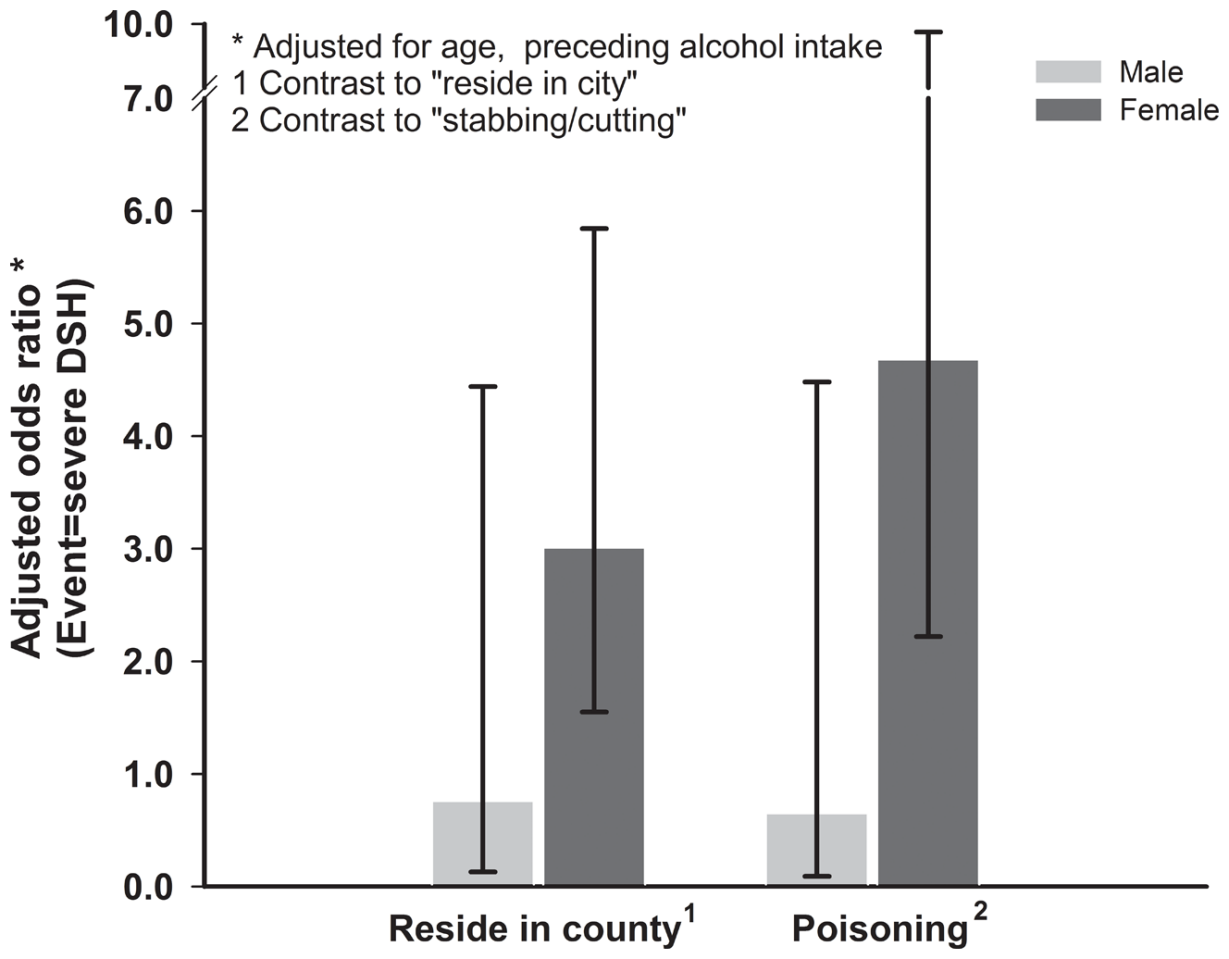
Gender-specific adjusted odds ratio of severe DSH by residence place and self-harm method, 2005-2010, Zhejiang, China.

Residence place and self-harm method showed gender-specific association with severe DSH. In female subjects, resided in counties was related to increased odds of severe DSH (adjusted odds ratio: 3.01, 95% CI: 1.55, 5.84), and compared with stabbing or cutting, poisoning was observed 4.68 (95% CI: 2.22, 9.87) times odds of severe DSH. However, in male subjects, both residence place and self-harm method were not significantly related to severe DSH (Figure 1).

## Discussion

In this study, we intended to explore associated factors of self-harm severity among DSH internal migrants in China. To our best knowledge, there are no existing literatures which ever addressed this topic before. Based on model fitting result, a positive association between age and severe self-harm has been identified. Considering of the intimate relationship between DSH and mental disorder, especially depression, such result may suggest that older migrants are more prone to be tortured by serious psychological problems. Some previous studies had pointed out that since the chance of permanently residing in host city is slim to most internal migrants, the majority of them will eventually choose to head back home at the rear of the productive age (usually from 50 to 60 years old), maybe in order to guarantee the full access to medical care (which is also determined by *hukou* status), in case of frequent ailments in the coming later age [14]. In this instance, migrants who still linger in host cities in their 50s or 60s are probably confronted with more serious financial predicaments, such reality can bring about a huge psychological pressure, which may adversely affect mental health. Moreover, internal migrants tend to leave home at a younger age, thus those older ones have already stayed in host cities for a considerably long period. In order to save living expenses as much as possible, most married internal migrants choose to live alone, longer separation from family can definitely do harm to mental health, too.

Although many existing literatures indicated that alcohol consumption is a major risk factor for suicidal behavior and DSH [15], [16], the association between alcohol involvement and DSH severity has seldom been discussed. One previous study suggested that as both DSH and alcohol use are risk factors for completed suicide, drinking may further increase the risk of impulsive behavior during DSH process, and cause aggravated injuries in turn [17]. However, our finding was quite in the opposite, as an inverse association has been detected between preceding alcohol drinking and severe DSH among study subjects. We think coping strategy theory can make a plausible explanation to this phenomenon: even if often be labeled as “maladaptive coping” or “avoidance coping”, alcohol drinking still can be an effective way of anguish venting, which is expected to mitigate the feeling of desperation among psychologically pressured individuals, and reduce the risk of severe DSH eventually.

In this study, we found that residence place showed significant association with severe DSH only among female subjects, such result may suggest that the psychological well-being of female internal migrants is more susceptible to social environment. Compared with city-dwelled counterparts, we think two major reasons may jointly contribute to higher odds of severe DSH among female internal migrants who reside in county. One is income difference, for job opportunities in cities are usually much higher paid than those in counties, so female migrants who reside in counties are in face of greater economic pressure, which can cause worse mental health. In addition, the mindset of city dwellers in China is usually more open and compatible, whereas local residents in small places are comparatively conservative and exclusive, so female migrants who reside in big cities may have a much better chance to integrate themselves into social environment, and confront less discrimination. Previous studies have found that both dysfunctional social connection and discrimination were significantly related to higher level of depression among Chinese internal migrants [18], [19], therefore, it is not surprising to find a deteriorated mental health status among county-dwelled female migrants.

Two hypotheses can be generated based on our study results, and need to be verified by future studies. One is that, as poisoning was positively associated with severe DSH only in females when compared with stabbing/cutting, we hypothesize that gender-related difference in poisonous substance choosing when performing self-harm behaviors may partly contribute to this discrepancy. The second is that, as we did not identify a significant association between gender and severe DSH, this finding may suggest that although it has been reported that the general mental health well-being of male internal migrants was much more worrying than their female counterparts [20], the proportion of gravely stressed individuals was not significantly different between two genders.

Those major findings above indicate that among Chinese internal migrants, older ones, and females who reside in counties may be more likely to commit severe self-harm behaviors, thus merit intense focus. In view of the catastrophic impacts of severe DSH to either family or the entire society, population-based surveys are imperatively needed, to broadly explore the overall mental health well-being, especially depression and its associated risk factors among Chinese internal migrants, in order to construct targeted intervention measures and effectively implement them as soon as possible.

Several limitations of this study should be noticed. First of all, to some research variables, the estimated 95% confidence interval of adjusted odds ratio was large, which means the sample size of certain subgroups was small, and that will definitely affect the precision of estimation in this study. Secondly, when constructing logistic regression model, the study population we used was DSH internal migrants, so to a certain extent, the associations we detected between self-harm severity and related factors can be somewhat different from the estimations had we studied the general population of internal migrants instead. But considering of the fact that DSH is still a rare event among general migrants, if we intend to find possible risk factors at population level, we might need an extremely large sample size to guarantee enough events, thus our method is definitely a more realistic and efficient way to perform preliminary screening. Thirdly, because of data limitation, in this study, we can not determine the place of origin for study subjects, considering the reported discrepancy in psychological well-being between rural-to-urban and urban-to-urban internal migrants in China [21], the associated risk factors of severe DSH can also be different between those two subtypes, thus should be further investigated. Finally, all DSH migrants we studied were collected from a localized area, although Zhejiang province is one of the major recipients of internal migrants in China, the generalization of our study results should be cautiously made.

## References

[1] Wen M, Fan J, Jin L, Wang GX (2010) Neighborhood effects on health among migrants and natives inShanghai, China. Health Place 16: 452–460. 20060767 10.1016/j.healthplace.2009.12.001

[2] Akay A, Bargain O, Zimmermann K (2012) Relative concerns of rural-to-urban migrants in China. J EconBehav Organ 81: 421–441.

[3] National Bureau of Statistics of China (2011) Major results of the 6^th^ national population census data. Available: http://www.stats.gov.cn/zgrkpc/dlc/yw/t20110428_402722384.htm. Accessed 2013 Jan.13

[4] Solinger D (1999) Contesting citizenship in urban China: Peasant migrants, the state, and the logic of themarket. Berkeley: University of California Press.

[5] Jian XH, Huang K (2007) Up-to-date investigation report on rural migrant workers in China. Chn Popu ResEnvi 17: 1–6.

[6] Hu XJ, Cook S, Salazar MA (2008) Internal Migration and Health in China. Lancet 372: 1717–1719. 18930533 10.1016/S0140-6736(08)61360-4 PMC7135200

[7] Qiu PY, Caine E, Yang Y, Chen Q, Li J, et al (2011) Depression and associated factors in internal migrantworkers in China. J Affect Disorders 134: 198–207. 21705089 10.1016/j.jad.2011.05.043 PMC3189449

[8] Mou J, Cheng JQ, Griffiths SM, Wong SY, Hillier S (2011) Internal migration and depressive symptomsamong migrant factory workers in Shenzhen, China. J Community Psychol 39: 212–230.

[9] Suominen K, Henriksson M, Suokas J, Isometsa E, Ostamo A, et al (1996) Mental disorders and comorbidityin attempted suicide. Acta Psychiatr Scand 94: 234–240. 8911558 10.1111/j.1600-0447.1996.tb09855.x

[10] Haw C, Houston K, Townsend E, Hawton K (2002) Deliberate self harm patients with depressive disorders:treatment and outcome. J Affect Disorders 70: 57–65. 12113920 10.1016/s0165-0327(01)00317-2

[11] Lam CS, Angell B, Tsang HW, Shi K, Corrigan PW, et al (2010) Chinese lay theory and mental illnessstigma: implications for research and practices. J Rehabil 76: 35–40.

[12] Bertolote JM, Fleischmann A, De Leo D, Wasserman D (2004) Psychiatric diagnoses and suicide: revisitingthe evidence. Crisis 25: 147–155. 15580849 10.1027/0227-5910.25.4.147

[13] Zhejiang Provincial Bureau of Statistics (2011) Major results of provincial data from the 10^th^ nationalpopulation census. Available: http://www.zj.stats.gov.cn/art/2011/5/6/art_165_121.html. Accessed 2013 Jan.14

[14] Xiang B (2007) How far are the left-behind left behind? A preliminary study in rural China. Popul SpacePlace 13: 179–191.

[15] Evans E, Hawton K, Rodham K (2004) Factors associated with suicidal phenomena in adolescents: asystematic review of population-based studies. Clin Psychol Rev 24: 957–979. 15533280 10.1016/j.cpr.2004.04.005

[16] Hufford MR (2001) Alcohol and suicidal behavior. Clin Psychol Rev 21: 797–811. 11434231 10.1016/s0272-7358(00)00070-2

[17] Ogle RL, Clements CM (2008) Deliberate self-harm and alcohol involvement in college-aged females: acontrolled comparison in a nonclinical sample. Am J Orthopsychiatry 78: 442–448. 19123765 10.1037/a0014325

[18] Lin DH, Li XM, Wang B, Hong Y, Fang XY, et al (2011) Discrimination, perceived social inequity, andmental health among rural-to-urban migrants in China. Community Ment Health J 47: 171–180. 20033772 10.1007/s10597-009-9278-4 PMC2891847

[19] Mao ZH, Zhao XD (2012) The effects of social connections on self-rated physical and mental health amonginternal migrant and local adolescents in Shanghai, China. BMC Public Health 12: 97. 22299776 10.1186/1471-2458-12-97 PMC3305514

[20] Wong D, He XS, Leung G, Lau Y, Chang YL (2008) Mental health of migrant workers in China: prevalenceand correlates. Soc Psychiatry Psychiatr Epidemiol 43: 483–489. 18398559 10.1007/s00127-008-0341-1

[21] Chen J (2011) Internal migration and health: Re-examining the healthy migrant phenomenon in China. SocSci Med 72: 1294–1231. 10.1016/j.socscimed.2011.02.016 21435765

